# From HIV to COVID-19, Molecular mechanisms of pathogens’ trade-off and persistence in the community, potential targets for new drug development

**DOI:** 10.1186/s42269-022-00879-w

**Published:** 2022-07-06

**Authors:** Antoine AbdelMassih, Abrar Sedky, Ahmed Shalaby, AlAmira-Fawzia Shalaby, Alia Yasser, Aya Mohyeldin, Basma Amin, Basma Saleheen, Dina Osman, Elaria Samuel, Emmy Abdelfatah, Eveen Albustami, Farida ElGhamry, Habiba Khaled, Hana Amr, Hanya Gaber, Ismail Makhlouf, Janna Abdeldayem, Jana Waleed El-Beialy, Karim Milad, Laila El Sharkawi, Lina Abosenna, Madonna G. Safi, Mariam AbdelKareem, Marwa Gaber, Mirna Elkady, Mohamed Ihab, Nora AbdelRaouf, Rawan Khaled, Reem Shalata, Rudayna Mahgoub, Sarah Jamal, Seif El-Din El Hawary, Shady ElRashidy, Sherouk El Shorbagy, Tony Gerges, Yara Kassem, Yasmeen Magdy, Yasmin Omar, Yasmine Shokry, Aya Kamel, Rafeef Hozaien, Nadine El-Husseiny, Meryam El Shershaby

**Affiliations:** 1 Pediatric Department, Pediatric Cardiology Unit, Faculty of Medicine, Cairo University Children Hospital, Cairo University, Kasr Al Ainy Street, Cairo, 12411 Egypt; 2 Internship Research Program (Research Accessibility Team), Faculty of Medicine, Cairo University, Cairo, Egypt; 3 Faculty of Dentistry, Cairo University, Cairo, Egypt; 4 Pixagon Graphic Design Agency, Cairo, Egypt

**Keywords:** COVID-19, OMICRON, Trade-off, Invasion–persistence, Virulence–transmissibility

## Abstract

**Background:**

On the staggering emergence of the Omicron variant, numerous questions arose about the evolution of virulence and transmissibility in microbes.

**Main body of the abstract:**

The trade-off hypothesis has long speculated the exchange of virulence for the sake of superior transmissibility in a wide array of pathogens. While this certainly applies to the case of the Omicron variant, along with influenza virus, various reports have been allocated for an array of pathogens such as human immunodeficiency virus (HIV), malaria, hepatitis B virus (HBV) and tuberculosis (TB). The latter abide to another form of trade-off, the invasion–persistence trade-off. In this study, we aim to explore the molecular mechanisms and mutations of different obligate intracellular pathogens that attenuated their more morbid characters, virulence in acute infections and invasion in chronic infections.

**Short conclusion:**

Recognizing the mutations that attenuate the most morbid characters of pathogens such as virulence or persistence can help in tailoring new therapies for such pathogens. Targeting macrophage tropism of HIV by carbohydrate-binding agents, or targeting the TMPRSS2 receptors to prevent pulmonary infiltrates of COVID-19 is an example of how important is to recognize such genetic mechanisms.

## Background

After the discovery of the SARS-CoV-2 variant, Omicron, that sporadically arose in November 2021, it was compared to the Delta variant that was discovered in May 2021 in terms of virulence and transmissibility. While Omicron has shown higher rates of transmission than the other variants, it showed less virulent symptoms with relatively mild disease. The Delta variant, however, has been associated with high virulence causing severe lower respiratory tract symptoms and reduced transmission rate (Kumar et al. 2021). This leaves us wondering; does COVID-19 agree with the trade-off hypothesis?

The trade-off theory states that for a pathogen to reach the maximum pathogenic fitness (i.e., reproductive numbers) a compromised level of virulence must be reached. This could be explained by the fact that a pathogen’s ability to spread is directly proportional to its ability to induce symptoms that favor the pathogen’s spread such as coughing and sneezing (i.e., it is directly proportional to virulence). However, up to a certain degree of virulence, a reduction in the spread of the pathogen could occur by the reduction in the duration of the infectious period or reducing contact between the host and other potential hosts. This could be seen with a reduction in the host’s physical level of activity or their death. Therefore, it is predicted that compromised moderate levels of virulence should be in sync with the pathogen’s evolution (Alizon et al. 2009a, b; Goldhill and Turner 2014; Sasaki et al. 2022).

This hypothesis was initially documented by Anderson and May and later modified by Ewald as an alternative to the acknowledged idea that pathogens always progress toward avirulence, or what was known as the avirulence theory (Bull and Lauring 2014; Seal et al. 2021).

Hence, an equation was reached based on the multiple factors that are thought to affect transmission potential. Transmission potential, *T*, is proportional to the product of precontact transmission (which is infectiousness), considered *p*, the contact rate with other carriers of the disease, *c* with infectiousness duration *d*, virulence *v* can impact the three latter factors in various ways. This all gives the proportionality equation:

$$T \sim p\left( v \right) \times c\left( v \right) \times d\left( v \right)$$

On the other hand, the type of virus infection and the virus-induced effects (i.e., its virulence) on cells are dependent on the virus, the cell type and species, and often the physiologic state of the cell. Infection is initiated in the presence of cells capable of supporting viral replication (McKay et al. 2020).

The trade-off of virulence to transmissibility can explain the evolution of acute infections but falls short when addressing chronic infections. Strains with shorter infectious periods and higher transmission rates tend to spread at a faster rate than strains with longer infectious periods and lower transmission rates. However, such strains are more prone to extinction as their population is very liable to severe fluctuations. This indicates the need for a larger community size to overcome such fluctuations and decrease the risk of extinction. As such, the persistence of these highly transmissible strains is harder than those with lower transmission rates which require a smaller community size to persist (Brouwer et al. 2019).

Hence, Grenfell's “invasion–persistence” trade-off theory states that a balance must be reached between a strain's infectious period and its transmission rate.

When acute immunizing pathogens maximize between-host transmission, thus inducing violent epidemic outbreaks, their short-term advantage at invasion may come at the expense of their ability to persist in the population over the long term. When the host population is broken into many small patches—selection prefers less acute pathogens, which persist longer within a patch and thereby achieve ameliorated between-patch transmission: the invasion–persistence trade-off dominates in this regime (Alizon et al. 2009a, b; Gu et al. 2021).

An important insight is that the most favorable life history may also rely on the size of the local host population through an appearing invasion–persistence trade-off. Particularly, evolution will gravitate to choose increasing acuteness to direct immunizing pathogens toward the brink of their extinction. Particularly, when transmission depends on the pathogen load in an inverse fashion or, more generally, when mechanisms acting at the level of the individual host restrain fitness—and then only in host populations above a threshold size—is robust persistence of highly acute pathogens possible (Fisher 2021; Walther and Ewald 2004).

King et al. developed a model explicitly linking within- and between-host dynamics to show that, depending on the shape of transmission’s dose–response curve, the invasion–persistence trade-off has indeed the potential to influence life history evolution through extinction-mediated group selection (King et al. 2009; Seal et al. 2021).

In epidemics, pathogens undergo consequent mutations in their genetic codes that render them more resistant to pressure as antibiotic administration or environmental factors, which in turn favors their persistence in hosts. This ultimately results in a phenotype that is persistent in the host and most probably goes unnoticed due to the mild symptoms which were not typical for the original strain.

This study aims to explore the molecular mechanisms and mutations of different epidemic-causing pathogens that attenuated their virulence and increased their transmissibility.

## Main body

### Molecular mechanisms of invasion–persistent trade-off

#### HIV

UNAIDS’ latest data publication states that more than 36.7 million people are currently living with HIV-1 worldwide, with 1.8 million newly infected individuals reported in 2016. The combination antiretroviral therapy (cART) has provided beneficial mechanisms in combating the HIV-1 replication, for instance, hindering the disease progression, decreasing the viral load and boosting T cell immune function. In addition to the previous mechanisms, c-ART effectively prevents transmission to uninfected individuals. Before the ART era, the benefits of latency were not fully understood but now during ART, we can say that latency has a substantial role in avoiding the extinction of HIV (Castro-Gonzalez et al. 2018).

Although patients with higher viral loads will be more infectious, it is well known that untreated, these individuals have a poorer prognosis and, hence, will have fewer lifetime opportunities for transmission (Alizon et al. 2010; Ta et al. 2022).

We find that, since both the duration of infection and infectiousness determine the opportunities for the virus to be transmitted, this puts forward a trade-off between these contributions to the overall transmission potential. An infection with intermediate virulence will produce more transmissions during the infectious lifetime because it optimizes the trade-off between the rate of transmission and duration of infection (Alizon et al. 2010).

One of the principal mechanisms, by which HIV-1 evades hosts cell responses and antiretroviral therapy is the ability to infect latent cellular reservoirs. One of the most overlooked latent cellular reservoirs is tissue macrophages. Macrophages, like CD4 + T cells, express CD4 and chemokine coreceptors CCR5 and CXCR4 on their cell surface allowing for HIV-1 susceptibility. Macrophages are influenced by dual-tropic and CCR5-tropic viral infection; however, factors that aid tropism are more elaborate than coreceptor usage. Specifically, macrophages express significantly lower levels of CD4, meaning macrophage-tropic viruses must have a high degree of affinity to CD4 to mediate fusion with the macrophage cell membrane (Hendricks et al. 2021).

HIV-1 evolution to enter and replicate in macrophages ends up in Env proteins which will expeditiously utilize low densities of CD4 found on macrophages for infective agent entry. This ability to use a coffee density of CD4 may be a key feature that distinguishes M-tropic viruses from the more typical R5 T-tropic viruses, which need high densities of CD4 for entry into target lymph cells. HIV-1 is often CCR5 victimization (R5) and T cell tropic (T-tropic), targeting memory CD4 T cells throughout acute and chronic infections. However, viruses can expand into different cell variants. Macrophage-tropic (M-tropic) HIV-1 variants have evolved to infect phagocytes, that have solely low levels of surface CD4. In comparison with T-tropic viruses, M-tropic viruses infect monocyte-derived macrophages (MDMs) on an average of 28-fold more efficiently, have exaggerated sensitivity to soluble CD4 (sCD4), and have proficient usage of low-density CD4 with more sensitivity to CD4 binding website antibodies. In addition to the prior findings, we tend to postulate that low-density CD4 usage and sensitivity to soluble CD4 (sCD4) are distinct criteria for macrophage reaction. Furthermore, CD4 entry phenotypes are more reproducible than the relative infection of monocyte-derived macrophages (MDMs) (Arrildt 2015).

#### Malaria

Another example of pathogen evolution under the combined pressure of host immune responses and antimicrobials is the mutated strains of falciparum malaria. The escalation of artemisinin resistance due to mutations in Plasmodium falciparum K13 has weakened antimalarial efficacy and burdens the global antimalarial elimination campaign. Mutation of the PfK13 propeller domain plays a role in artemisinin resistance; also, it includes divergent mechanisms based on a quiescence state leading to parasite rejuvenation as soon as drug pressure is removed. Transcription analysis demonstrated an evident downregulation for 10 genes following exposure to DHA, but continued transcription of 2 genes encoding mitochondrial and apicoplast proteins. Transcription of various genes encoding mitochondrial and apicoplast proteins, especially genes encoding enzymes in pyruvate metabolism and fatty acid synthesis pathways, was also preserved. Supplementing inhibitors for biotin acetyl-coenzyme A (CoA) carboxylase and enoyl-acyl carrier reductase of the fatty acid synthesis pathways have slowed down the rehabilitation of stagnant parasites by 6 and 4 days, respectively, following DHA treatment. The results portray that majority of metabolic pathways are downregulated in DHA-induced dormant parasites. In opposition, pyruvate and fatty acid metabolic pathways remain functioning (Mok 2021).

#### HBV

One of the viruses that are also subject to the same type of viral trade-off is the hepatitis B virus. The high error rate of HBV reverse transcriptase (RT) was associated with developing several mutations. Although these mutants have errors in the replication or infectivity of the virus compared to wild-type HBV, they can turn out to be the dominant species due to their different response to the antiviral immune response or therapy. HBV genotypes, sub-genotypes and mutations in certain regions of the HBV genome have been found to influence many aspects of the virtual infection. Some of the adverse outcomes are linked to alterations in the pre-S/S region, for instance, occult HBV infection, vaccine malfunction and the occurrence of hepatocellular carcinoma (HCC). Moreover, mutations in the P region may cause the drug resistance to NA antivirals. Mutations in the pre-C/C region are related to HBeAg negativity, immune escape and persistent hepatitis (El-Mokhtar 2021; Yano 2015).

##### Pre-S/S mutations

Variants within the HBV surface antigens are correlated with occult HBV infection (OBI) which is defined as the presence of HBV DNA in the liver tissue of HBsAg-negative individuals.

Deletions in the pre-S region can lead to decreased expression of HbsAg, hence its absence in the serum. It also aids in viral persistence by removing HLA-restricted B cell and T cell epitopes. The overlapping between the genes encoding reverse transcriptase (RT) and HBsAg causes differences in the major hydrophilic region of HBsAg that reduce the binding affinity of neutralizing antibodies, including those induced by HBV vaccine. Immune-associated escape mutations lead to harder HBsAg recognition by vaccine-induced antibodies. In addition to this, immune-associated escape mutations can lessen HBsAg binding to antibodies used in diagnostic assays for HBsAg detection and quantification which therefore results in false negativity or an underestimation of HBsAg levels (Glebe et al. 2005; Alizei et al. 2021).

##### P Region Mutations

P region encodes for reverse transcriptase (RT) enzyme. Mutations in RT can result in drug resistance after long-term use of nucleotide analogues (NA). Antiviral drug resistance can be related to the selection of adaptive mutations which decreases the sensitivity of the mutants to the inhibitory effects of a drug.

In addition to NA-induced escape mutations, 29 immune-associated escape mutations were found in the major hydrophilic region of HBsAg. Studies show that certain circulating HBV strains have a higher number of immune-associated escape mutations than others; for instance, genotype D has more mutations than A (Lazarevic et al. 2019; Zoulim and Locarnini 2009).

##### Pre-C/C region mutations

The commonest precore (PC) mutation terminates HBeAg expression at the translational level. Despite the previous common belief that HbeAg acts as tolerogen and helps the immune escapism of HBV, it is now increasingly recognized that it acts as an immunogen, enhancing the immune clearance of HBV. The HBeAg-negative cases who undergo HBeAg seroconversion demonstrate a recurrence of high HBV DNA levels unlike HBeAg-positive cases, where seroconversion is accompanied by decreased HBV DNA levels. This renders HBeAg-negative cases to exhibit unsuccessful immune clearance. Consequently, HBeAg-negative chronic hepatitis B is currently the leading type worldwide as well as the most difficult in treatment regarding achieving sustained virological response. Mutations in HBcAg may result in the formation of immune escape variants, leading to the persistence of HBV. The core protein HBcAg of HBV is a potent immune stimulator, stimulating a strong neutralizing immune response mainly in the form of the HBV-specific CD8 + T lymphocytes (CTL)-mediated immune response. The more conserved the HBV core protein (HBc), the stronger the CTL responses. This suggests that the HBc-specific T cell response may play a leading role in viral control and clearance (Colagrossi 2018).

#### Tuberculosis

Mycobacteria escape the host immune defense mainly by staying dormant (Salina et al. 2019). Dormancy passes through three stages in which TB forms an asymptomatic latent infection that may last for many years. The first stage, termed the non-replicating persistent stage (NRP-1), has an increased culture medium turbidity, 10% oxygen level, and gradual rise in antibiotic tolerance. In the second stage, there is no more increase in the medium turbidity and the cells show an arrested growth in the cell cycle (NRP-2). Lastly and only in (L4) lineage, a viable but non-cultivable state (VBNC) is developed after 3–4 weeks in the dormancy state. VBNC can shut down the major replicative and metabolic activities by regaining their activity after oxygen reintroduction. Dormancy explains the fact that TB infection can persist in the hosts and escape immunity for long periods (Peddireddy et al. 2017; Batyrshina and Schwartz 2019).

Researchers showed that the growth rates upon hypoxia-induced dormancy were similar among lineages, L1, L2 and L3. However, only lineage 4 recovered growth and metabolic activity upon providing oxygen and fresh media, even after prolonged periods of hypoxia. The anaerobic NRP-2 phase as compared to lineage “two” was able to survive only relatively shorter periods of induced hypoxia. Lineage influences dormancy survival. Only strains belonging to the Euro-American (L4) lineage rebounded from O2 deprivation, but strains belonging to others did not (L1, L2, L3) (Tizzano 2021).

This peculiar ability of L4 is due to the upregulation of the dormancy survival regulator gene (DosR). The DosR regulon allows the pathogen to withstand hypoxia. Transcriptomic analysis for the experimented lineages showed upregulation of DosR genes in L4. This explains its survival during oxygen deprivation. Four hundred seventy-one genes were upregulated and 483 downregulated with a fold change of at least 2, including 36 induced (out of 48) DosR regulon genes. The Beijing (L2) strain had a different expression pattern than H37Rv (L4) (Chen et al. 2013; Dartois and Rubin 2022).

### Acute infections abiding by virulence–transmissibility trade-off

#### Influenza

Hemagglutinin (HA), a surface glycoprotein, initiates influenza infection. It binds and fuses viral and endosomal membranes using sialic acid-containing molecules as receptors. In an experiment, all human influenza virus strains readily attached and decorated ciliated epithelial and goblet cells from human upper respiratory tract tissues, such as the nasal septum, concha and nasopharynx (de Bruin et al. 2022; Kumlin et al. 2008). In contrast, avian influenza viruses bound poorly to the same tissues. The same group of researchers has previously shown that avian influenza viruses can bind well to cells in tissue sections from the human lower respiratory tract, especially to type II pneumocytes and alveolar macrophages. Influenza viruses, on the other hand, do not bind to macrophages and type II pneumocytes. The discrepancy in tissue tropism of highly virulent avian influenza strains such as H5N1, which are less transmissible between humans and low pathogenic seasonal human influenza strains, can only be understood by the distribution of sialic acid in the human airway. SAα2,3 occupies alveolar cells, while SAα2,6 non‐alveolar cells. SAα2,3 and SAα2,6 distribution correlate with lower respiratory tract symptoms and difficulty ascertaining H5N1 in nasopharyngeal samples (Ayora-Talavera 2018). Mutations in HA can modify the tissue tropism of influenza and subsequently influence its transmissibility and virulence. Tumpey and colleagues compared the virulence and transmissibility of three variants of the 1918 influenza virus: the South Carolina strain (SC18), the Avian 1918 strain (AV18) and the New York strain (NY18). NY18 strain was both the most virulent (18.7% weight loss) followed by AV18 (14%), and SC18 was relatively less virulent with only 11% weight loss in ferrets. NY18 harbored mutations of its HA receptors rendering it able to bind to both types of sialic acids, while AV18 could only attach to SAα2,3, making it highly virulent but nearly non-transmissible between ferrets. This is a classic example of how genetic mutations of viral receptors can mediate the transmissibility–virulence trade-off (Dorna et al. 2022; Tumpey et al. 2007).

#### COVID-19 Omicron variant, why is it more transmissible yet less virulent? Is it a classic illustration of the trade-off theory?

Reports from South Africa, Scotland and England showed lower hospitalization rates following Omicron infection than the Delta variant; several studies support this (Wang et al. 2022).

A study conducted in 499 hospitals in South Africa reported 21 (4.5%) hospital deaths during the Omicron wave, compared to 847 (21.3%) deaths during the Delta wave; it also showed that hospital admissions were 1% of cases during the Omicron wave and 4.3% during previous ones; the duration of hospital stay itself was also shorter in the Omicron wave (4 days) than in previous waves (8.8 days) (Abdullah et al. 2022).

Another study compared 3-day risks of emergency department visits, hospitalization, ICU admission and mechanical ventilation in patients infected with both Omicron and Delta variants. Every studied aspect was lower with Omicron than with Delta. Emergency department visits were 10.67% fewer during Omicron, hospitalizations 2.2% lower, ICU admissions 0.52%, and mechanical ventilation 0.36% less (Wang et al. 2022).

The UK's health security agency and Cambridge University's biostatics unit reported similar findings. They compared 6312 Omicron patients with 8875 Delta patients. It is revealed that 21 Omicron patients (0.3%) and 116 (2.2%) Delta patients were hospitalized. There were no confirmed deaths in matched Omicron-infected patients, but 7 (0.3%) deaths in Delta-infected patients. In addition, the risk of hospitalization or death was 68% lower in Omicron cases than in Delta cases. The risk of hospitalization or death was also 54% lower in vaccinated Omicron cases than in vaccinated Delta cases though more infected patients (Mohapatra et al. 2022). The Omicron variant is more infectious with fewer repercussions (El-Shabasy et al. 2022a, b, c, d). In the coming sections, we will explain the varied disease presentations.

The omicron variant did not directly evolve from a previously known variant. A possible scenario is that the precursor of the omicron virus jumped from humans to mice, where it mutated and then transferred back into humans (Wei et al. 2021).

Fantini et al. conducted a structured analysis of the Omicron variant. Omicron showed 30 spike protein mutations, three deletions and one insertion. For comparison, Delta B.1.617.2 has nine mutations and one deleted part. The mutations heavily affected the receptor-binding site (RBD), the N-terminal domain (NTD) and other regions such as the proteolytic cleavage site and the fusion machinery (Fantini et al. 2022).

Owing to these mutations, the affinity of the spike protein to ACE2 receptors has increased.

SARS-CoV-2 gains entry to host cells primarily via the binding of the spike (S) protein and the angiotensin-converting enzyme II (ACE2). This binding leads to either the endolysosomal (endocytic) or the TMPRSS2-mediated entry. The endocytic route is pH-sensitive. The acidic endosomal pH activates the virions, which then fuses the viral and endosomal membranes and releases the viral genome into the cytosol. The endocytic pathway also includes caveolae-dependent endocytosis, macropinocytosis and Clathrin-dependent endocytosis (Jackson et al. 2022).

On the contrary, recent studies have indicated that the TMPRSS2 drives a pH‐independent entry of SARS‐CoV‐2. Serine proteases at the plasma membrane—including transmembrane protease serine 2—cleave the spike protein. The genetic material thus enters into the cytosol (Koch et al. 2021).

The Delta and Omicron variants use different pathways, which affect their replication efficiency. Zhao et al. (2022) and Peacock et al. (2022) tested this by infecting TMPRSS2 expressing VeroE6 cells and Calu3 cells or non-TMPRSS2-expressing parental VeroE6 cells with Omicron or Delta variants (Peacock et al. 2022; Zhao et al. 2022). Viral RNA copies were subsequently measured. Results showed that in TMPRSS2 expressing cells, the Omicron variant had significantly lower viral loads than the Delta variant had. Although both VeroE6/TMPRSS2 and Calu3 exhibit high levels of TMRPSS2 expression, the Omicron variant replicated differently between these two cell lines. Omicron relies heavily on endocytic entry, while Calu3 cells do not allow that. Omicron is less efficient than the Delta variant in cells expressing TMPSSR2. To prove this, researchers compared Omicron to Delta. They used endocytosis inhibitors (such as bafilomycin A1 and chloroquine) and a TMPRSS2 inhibitor (Camostat). Bafilomycin A1 and chloroquine inhibited Omicron and Delta variant viral replication in VeroE6/TMPRSS2 cells. In contrast, the inhibition by Camostat only affected the Delta variant. These results show that the Omicron variant is dependent on the endocytic pathway and not the TMPRSS2 path for viral replication (Jackson et al. 2022).

Another fundamental finding of this study is that Omicron outcompetes Delta in primary cultures of the human nasal airway epithelial cells (hNECs) (Zhao et al. 2022). They mixed a competition assay with an equal amount of each variant into the same wells of hNECs, Vero-ATs or Calu-3 cells. The Omicron was the only virus yielding detectable RNA products in hNECs (Table 1).

**Table 1:** Summary of the main molecular trade-off mechanisms favoring transmissibility/latency over virulence

	Main molecular mechanism	Less virulent strain	Drugs under trial	More virulent strain	References in text
Acute infections: Virulence trade-off theory					
Influenza	Mutations in HA favoring non-alveolar cell tropism by differential sialic acid tropism	Seasonal human influenza strains	Umifenovir inhibits viral attachment to sialic acid	Avian influenza strain H5N1	Popov et al. (2021)
COVID-19	Mutations in spike protein promoting endocytic rather than TMPRSS2 mediated entry impeding lower respiratory tract infection	Omicron variant	TMPRSS2 inhibitors such as CAMOSTAT	Delta variant	Shapira (2022)
Chronic infections: Invasion–persistence theory					
HIV	Promoted sensitivity to CD4 mediated fusion into latent cellular reservoirs thus escaping retroviral therapy	M-tropic viruses	BIT225 PI3K/AKT inhibitors	R5 T-tropic viruses	Pasquereau and Herbein (2022)
Malaria	Mutations in PfK13 allows survival	Artemisinin- resistant Plasmodium falciparum strains	Targeting cyclin dependent kinases in Malaria to overcome drug resistance and dormancy	Non artemisinin resistant Plasmodium falciparum strains	Balestra et al. (2020)
HBV	Mutations in pre-S/S region promoting vaccine failure, immune escape, occult HBV infection and generation of HCC	Mutated HBV strains	Checkpoint inhibitors and therapeutic vaccines to overcome immune escapism of the new variants of HBV	Wild-type HBV	Hoogeveen and Boonstra (2020)
	Mutations in the P region encoding for RT promoting drug resistance to NA antivirals				
	Mutations in pre-C/C region prompting HBeAg negativity via termination of its translation, immune escape and HBV persistence				
Tuberculosis	DoSr upregulation allowing survival in prolonged hypoxia	L1, L2 & L3 lineages	The carbonic anhydrase inhibitor ethoxzolamide	L4 lineage	Aspatwar et al. (2018), Zheng and Abramovitch (2020)

*AKT Aromatase*, *HBV* hepatitis B virus, *HIV* human immunodeficiency virus, *R5* CCR5 victimization, *M-tropic* macrophage-tropic viruses, *T-tropic* T cell tropic, *PfK13* Plasmodium falciparum K13, *PIK* phosphoinositide 3-kinases, *RT* reverse transcriptase enzyme, *NA* nucleoside analogues, *PC* precore, *DoSr* dormancy survival regulator gene, *TB* tuberculosis

As aforementioned, mutations of the spike protein (mutations on the amino acid sequence and surface electric charges) (Fantini et al. 2022) led to a reduced binding ability to the TMPRSS2-mediated cleavage system at the plasma membrane of the respiratory tract cells. TMPRSS2 is expressed intensively in alveoli and poorly in upper respiratory tract cells. Hence why the omicron variant minimally affects the lower respiratory tract.

Some factors affect the endolysosomal pathway of viral entry to the cell and are different in the upper and lower respiratory tracts. Firstly, the temperature has a significant contribution. Pastuszka et al. (2014) describe switching off cellular pathways using thermally responsive protein polymers (Pastuszka et al. 2014). A minimal increase in temperature stimulates cytosolic elastin-like polypeptides (ELPs) to fuse with the so-called Clathrin light chain, the main protein mediating endocytosis. This fusion contributes to the switch-off of the endolysosomal path of cell entry.

**Fig. 1:**
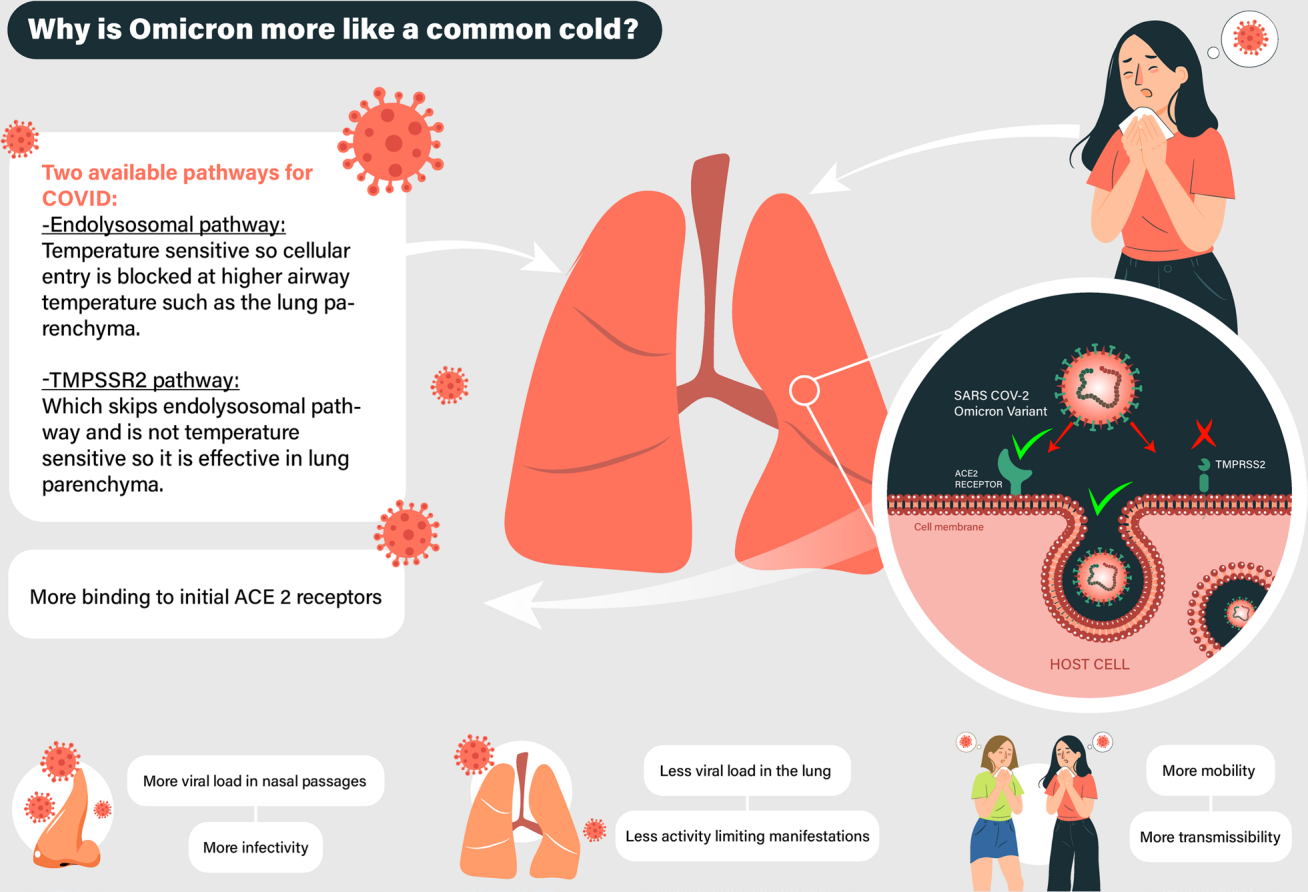
Why is Omicron more like a common cold? SARS-CoV-2: severe acute respiratory syndrome coronavirus 2, TMPSRR2: transmembrane serine protease

Vacuolar acidification is the second factor significant for the infectious entry process. Janicka-Russak et al. studied the effect of low temperature on modification of the plasma membrane H + -ATPase activity in cucumber roots. They revealed that plasma membrane H + -ATPase activity decreased in plants treated for 3 days with lower temperatures (Janicka-Russak et al. 2012). Based on these findings, we can conclude that Omicron replication is more effective in the upper airway because the lower temperature maintains a low endolysosomal pH. The pH switches on the endocytic pathway, resulting in enhanced transmissibility of the omicron variant. Figure 1 summarizes the aforementioned mechanisms (Su 2021).

#### Vaccination an accelerator of trade-off theory?

As stated above, the viral trade-off theory states that an intermediate degree of viral virulence is needed to optimize viral transmission. If a pathogen were too virulent, it would decrease the contact and duration of infectiousness. Hence, it would lower transmissibility. COVID-19 vaccines participate in this viral evolution.

One study by Indiana University School of Medicine in the USA showed that 64% of vaccinated people infected with COVID-19 had only asymptomatic infections. This study shows that the use of vaccines is pivotal in reducing severe diseases requiring hospital admission, therefore reducing mortality rates from COVID-19 (McKay et al. 2020).

Thanks to vaccines, we now see milder to asymptomatic infections. However, during the spread of the Delta strain, vaccines were hypothesized to have a limited role in reducing viral transmission. There was a significant rise in infections in vaccinated individuals.

A study by Anika Singanayagam and her colleagues shows that the rate of SARS-CoV-2 transmission is very similar when comparing vaccinated and unvaccinated people. They tested peak viral titers in upper respiratory passages and found similar levels in unvaccinated and vaccinated infected individuals, even those with asymptomatic infection (Franco-Paredes 2022).

Less severe infections among vaccinated individuals would translate to decreased virus virulence and increased patient mobility. Increased contact and community transmission of higher viral loads would thus occur (Singanayagam 2021; Zhang et al. 2022). Consequently, we could achieve herd immunity faster.

#### Can trade-off hasten reaching herd immunity?

Indirect protection from a transmissible infectious disease occurs when a population receives immunity through mass vaccination or previous infection.

In October 2020, some epidemiologists from the UK and USA recommended an approach they termed “focused protection.” It entails easing the restriction on low-risk groups, to allow them to establish immunity to SARS-CoV-2 through natural infection, while simultaneously stepping up the protection of high-risk groups. However, the opposition defended the restrictions done to slow the spread of the virus to reduce drastic consequences (Burki 2021). Acquiring herd immunity naturally rather than through vaccination can be very challenging due to the high rate of severe illness and death. Hospitals would fill up beyond their surge capacities. In overpopulated countries like India, exposing the population to COVID-19 to acquire herd immunity may prove disastrous and fatal. Therefore, vaccines are safer for achieving herd immunity.

What might have been too risky then might prove to be a key strategy now because of the current *less severe Omicron variant* and the abundant vaccinations.

Even after the availability of vaccines, the possibility of achieving herd immunity is difficult where ongoing mutations of SARS-CoV-2 result in an increased spread, and relative evasion of neutralizing antibody activity caused by previous virus infection or COVID-19 vaccines is likely (Garg et al. 2021). But the viral trade-off theory might be the key to achieving it.

## Conclusions

It is clear from the above that random mutations can mediate virulence–transmissibility trade-off in acute infections, and virulence (invasion)–persistence trade-off in chronic infections. These mutations can be a great potential for drug development. Several new drugs are under development, that are a good illustration of the exploitation of these molecular targets. For chronic infections, targeting cyclin-dependent kinases that mediate dormancy in malaria, or the use of ethoxzolamide in TB is under trial. Blocking HIV entry into macrophages by aromatase inhibitors has been recently FDA-approved. Inhibitors of viral proteases mediating lung entry of SARS-CoV-2 and influenza attachment to sialic acid are also new potential targets for drug development (Aspatwar et al. 2018; Balestra et al. 2020; Hoogeveen and Boonstra 2020; Pasquereau and Herbein 2022; Popov et al. 2021; Shapira 2022; Zheng and Abramovitch 2020).

## Data Availability

Not applicable.

## Abbreviations

ACE2: Angiotensin-converting enzyme
AKT: Aromatase
ATP: Adenosine triphosphate
BIT: Biotron Limited
c-ART: Combined antiretroviral therapy
CCR: Chemokine receptor
CD: Cluster of differentiation
COVID-19: Coronavirus disease 2019
DHA: Dihydroartemisinin
DosR: Dormancy survival regulator gene
FDA: Food and Drug Administration
HA: Hemagglutinin
HBcAg: Hepatitis core antigen
HBsAg: Hepatitis B surface antigen
HBV: Hepatitis B virus
HIV: Human immunodeficiency virus
ICU: Intensive care unit
NRP: Non-replicating persistent
PFK13: Plasmodium falciparum Kelch 13
PIK 13: Phosphoinositide 3-kinases
PreC: Pre-Core
PreS: Pre-surface
RNA: Ribonucleic acid
RT: Reverse transcriptase
SA: Sialic acid
SARS-CoV-2: Severe acute respiratory syndrome causing coronaviridae 2
TB: Tuberculosis
TMPRSS2: Transmembrane serine protease 2
UK: United Kingdom
UNAIDS: The Joint United Nations Programme on HIV/AIDS
USA: United States of America
VBNC: Viable non-cultivable
